# Prediction of gene expression regulation by human microRNAs in *Plasmodium falciparum*

**DOI:** 10.1186/s41021-021-00198-y

**Published:** 2021-06-15

**Authors:** Alexandr Grinev, Natalya Fokina, Denis Bogomolov, Iza Berechikidze, Yuliya Lazareva

**Affiliations:** Department of Biology and General Genetics, I.M. Sechenov First Moscow State Medical University (Sechenov University), Moscow, Russia

**Keywords:** Bioinformatics, Falciparum, Malaria, microRNA, Plasmodium, RNA interference

## Abstract

**Background:**

Malaria is a disease annually causing over 400,000 deaths. Deep understanding of molecular and genetic processes underlying its life cycle and pathogenicity is required to efficiently resist it. RNA interference is a mechanism of the gene expression regulation typical for a wide variety of species. Even though the existence of this phenomenon in *Plasmodium falciparum* has long been rejected, several recent works pose hypotheses and provide direct and indirect evidence of the existence of mechanisms similar to RNA interference in this organism. In particular, the possibility of regulation of *P. falciparum* gene expression through human microRNAs is of great importance both for fundamental biology and for medicine. In the present work we address the problem of possibility of the existence in the *P. falciparum* genome of the nucleotide sequences such that mRNAs transcribed from genes containing these sequences could form duplexes with human microRNAs. Using bioinformatics methods we have analysed genomes of 15 *P. falciparum* isolates for sequences homological to these microRNAs.

**Results:**

The analysis has demonstrated the existence of a vast number of genes that could potentially be regulated by the human microRNAs in the plasmodial genome.

**Conclusions:**

Despite the fact that the numbers of homological intervals vary significantly between isolates, the hsa-miR-451a and hsa-miR-223-3p microRNAs are expected to make the most notable contribution to the pathogenesis of *P. falciparum* malaria. The majority of homological intervals occur in genes encoding cell adhesion proteins.

## Introduction

RNA interference is one of the most prominent breakthroughs in molecular genetics of the late twentieth century. In the broad sense of the term, the essence of this phenomenon lies in eukaryotic gene expression regulation (in most cases, their silencing) by small non-coding RNAs (ncRNAs). Initially the use of the term was limited to posttranscripional gene silencing (PTGS) mediated by small non-coding RNAs of a certain class called small interfering RNAs (siRNAs). In this case, gene silencing occurs due to the formation of complementary bonds between a siRNA and a target mRNA either leading to the endonuclease clevage of the target RNA or directly impeding its translation. Further research has revealed the existence of several other types of small ncRNAs having similar mechanisms of functioning, as well as other mechanisms of gene expression regulation mediated by small non-coding RNAs. To date, the most extensively studied class of small non-coding RNAs participating in such interactions is microRNA, which are double-stranded RNA molecules typically from 19 to 25 nucleotides in length. Unlike siRNAs, microRNAs are endogenous and can utilise various mechanisms to regulate gene expression: besides the endonuclease cleavage of the target mRNAs, a microRNA can cause translation termination during initiation or elongation. MicroRNA and siRNA also differ in the anticipated function: microRNAs are considered to alter gene expression, stabilise mRNA and protein level and repress stochastic fluctuations of the gene expression level between different cells while siRNAs predominantly target exogenous nucleic acids such as mobile genetic elements and viral mRNA [1].

To date, a large amount of genetic data has been accumulated both for humans and *Plasmodium falciparum*, which causes the most severe forms of malaria, including the genomes of various strains and isolates of the parasite. Existing bioinformatics methods and tools allow to process genome sequences with reasonable accuracy.

Thus, the analysis of the *P. falciparum* genome is required in order to identify potential interactions of its mRNAs with regulatory microRNAs, as well as to compare the genomes of its various strains to determine the level of their pathogenicity due to the differences in these interactions.

**Fig. 1:**
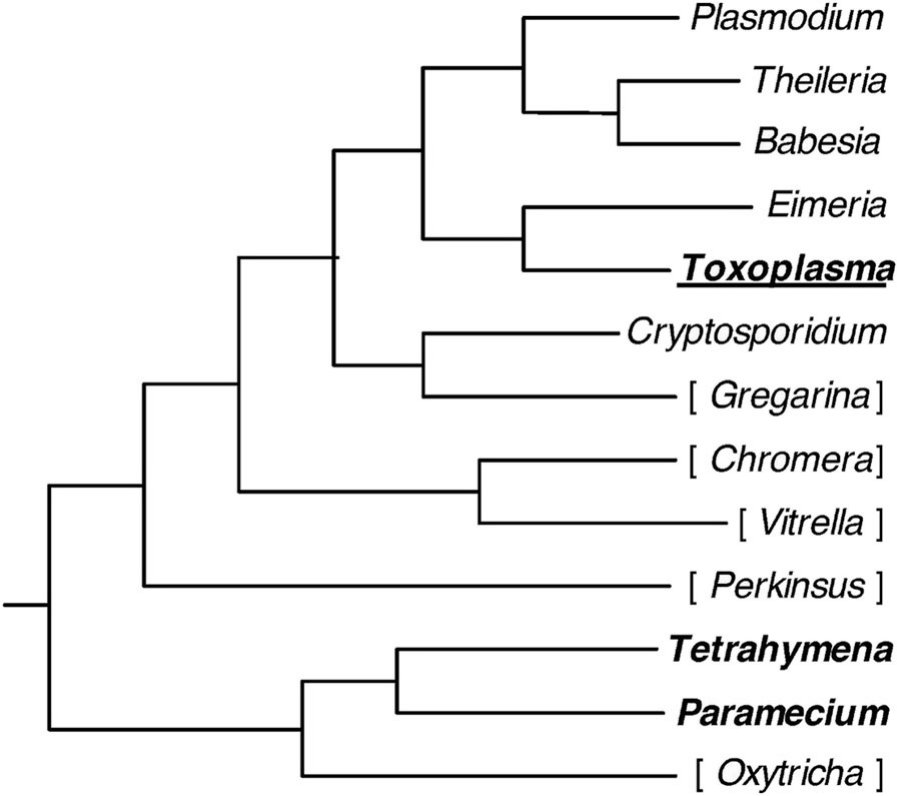
Phylogenetic tree of the *Alveolata* superphylum. The phyla for those the existence of an RNA interference pathway has been demonstrated are denoted in bold. The only known phylum of alveolates – *Toxoplasma* – is known to possess genes encoding microRNAs and the components of the RISC; for this reason its name is underlined. The names of the phyla for which corresponding studies are lacking are given in brackets. Adapted from Fig. 1 in [9]

The phenomenon of parasite gene silencing by small non-coding RNAs of the host organism is known to be present in several species of *Eukarya* [2–4]. In turn, the *Plasmodium* genome lacks genes encoding both microRNAs and proteins required for their processing and for proper functioning of the RNA-induced silencing complex (RISC) which mediates the target mRNA degradation or translation termination [5–7]. Furthermore, RNA interference has been observed only in one species belonging to the *Apicomplexa* genus, *Toxoplasma gondii* [8]. Neither a functioning RNA interference pathway nor genes encoding proteins involved in it have been discovered in phylogenetically more close to *P. falciparum* genera *Babesia* and *Theileria*, which belong to *Piroplasmida* order. This order forms a monophyletic group with the *Haemosporida* order, containing the *Plasmodium* genus, and a paraphyletic one – with the *P. falciparum* itself [9]. The phylogenetic tree of the *Alveolata* superphylum with the highlighted phyla known to possess either a functioning RNA interference pathway or genes encoding microRNAs is shown in Fig. 1.

Nevertheless, there is multiple indirect evidence supporting the hypothesis that human microRNAs might influence the expression of specific genes in *P. falciparum*. In a similar manner, the change of the microRNA level in the vector and definitive host of the parasite, the *Anopheles spp.* mosquito, has been observed to induce changes in the stage of the life cycle of the parasite, which may act as a defence mechanism of the mosquito [10].

In 2012 LaMonte et al. [11] discovered that human microRNAs are able to penetrate the *P. falciparum* cell and form duplexes with its mRNAs, which, in turn prevents these mRNAs from translation. Furthermore, this phenomenon can be treated as another reason explaining low susceptibility of people suffering from the sickle-cell anaemia to malaria, since their erythrocytes contain abnormal amount of miR-451 and miR-223. Previously this phenomenon was explained predominantly by the low nutritional value of the HbS haemoglobin for the parasite [11]. Despite the fact that the mechanism the existence of which has been hypothesised in this work does not require the involvement of any RNA inteference molecular machinery, it has got a considerable amount of similarity with the kind of RNA interference typical for the majority of animals, i.e. translation termination caused by the formation of duplexes consisting of a microRNA and its target mRNA.

In the succeeding work by Dandewad et al. [12] it has been observed that elements of the RISC, in particular, the Argonaute 2 protein, can be transported into the parasite together with the microRNA. An important role in this process is played by so called exosomes, i.e. small vesicles surrounded by lipid bilayers the diameters of which range from 50 to 300 nm [6, 13–15]. Most of these vesicles are synthesised within thrombocytes, but erythrocytes, leucocytes and endothelial cells can also produce exosomes [6, 13]. Mantel et al. [16] have demonstrated that erythrocytes infected with *P. falciparum* secrete vesicles containing nucleoprotein complexes consisting of microRNAs and Argonaute 2 proteins. Moreover, vesicle produced by the infected erythrocytes contain proteins of the RBCs, plasmodial proteins and small RNAs [17] including human tRNAs, Y-RNA, vaultRNA, snoRNA, piRNA and RNAs of *P. falciparum*, in particular, mRNAs encoding exported proteins, proteins inducing drug resistance in the parasite [18], and ncRNAs: rRNAs, snRNAs and tRNAs [19]. Exosomes secreted by the invaded cells contribute to the transition of *P. falciparum* to the subsequent stages of its life cycle, in particular, to the development of gametocytes [17].

Moreover, exosomes derived from the invaded red blood cells influence other cells in human body. The succeding uptake of these vesicles by endothelial cells leads to the alteration of gene expression in them, and as a result, to the change in the barrier properties of these cells. Thus, an increase in the permeability of the walls of blood vessels can occur both due to the direct interaction of the infected red blood cells with endothelium and indirectly, through a mechanism similar to trogocytosis, i.e. via exosomes. Furthermore, exosomes are endocytosed by macrophages and red blood cells. In case of endocytosis of vesicles by macrophages, the latter are activated, which leads to the production of pro-inflammatory cytokines, namely, interleukin-6 (IL-6) and interleukin-1 (IL-1), and entails a decrease in the endothelial barrier function and an increase in the number of adhesion molecules on the surface of these cells. In particular, during the endocytosis of the vesicles by endothelial cells, the appearance of VCAM-1 molecules on their surface is observed [16]. Besides, it has been found that exosomes secreted by red blood cells infected with *P. falciparum* trophozoites are absorbed by monocytes significantly more actively than those originating from healthy red blood cells [15].

Normally, human discocytes contain microRNAs of 21 different types as well as Ago2 proteins. Together they form the core of the RISC. An important feature of these microRNA/Ago2 complexes is their high stability in the absence of target mRNAs. Dicer and Drosha proteins required for the maturation of microRNAs, are present in young red blood cells, but are lost as they differentiate. A significant decrease in the levels of all microRNAs, with the exception of miR-451a and let-7b, is observed in the invaded RBCs.

Quantitatively, miR-451a constitutes the largest proportion of total amount of microRNA in both healthy and infected red blood cells. The primary role of this microRNA is considered to be the regulation of erythropoiesis. The targets for miR-451a include the mRNAs encoded by the CAV-1 and ATF2 genes. The CAV-1 gene encodes the protein caveolin-1, which is involved in the process of endocytosis; ATF2 encodes the self-titled transcription factor expressed in large quantities in healthy endotheliocytes. This microRNA is exported via exosomes, which leads to a significant decrease in the amount of its target mRNAs, thus altering the functional characteristics of endotheliocytes [16].

The aim of this study is to identify potential interactions of its mRNAs with regulatory microRNAs, as well as to compare the genomes of its various strains to determine the level of their pathogenicity due to the differences in these interactions.

## Materials and methods

We used blastn from the BLAST+ 2.9.0 package [20] to locate sequences encoding mRNAs which possibly interact with the selected human microRNAs. The analysis was performed on a 3.0 GHz Intel Pentium 4 computer with 3.0 Gb of RAM under Ubuntu Linux 10.04 × 86. We used human microRNA sequences from the miRBase database (release 22) and genome sequences of various *P. falciparum* isolates from PlasmoDB (release 45) and the database of the Sanger Institute [21–23].

### Target microRNA sequence generation

One of the steps in the microRNA target prediction was the transformation of the analysed microRNAs, since the input data in our analysis consisted of a set of microRNA sequences and a set of *P. falciparum* genomes. Interactions between a microRNA and its target mRNA follow the principle of complementarity. Due to this fact, the target mRNA should contain a subsequence complementary to the inverted nucleotide sequence of the microRNA, as the latter is antiparallel to the target mRNA.

Since the genome is represented by the DNA sequences, it was necessary to identify its regions from which the target mRNA were transcribed. As a rule, a mRNA is transcribed from the antisense strand and therefore it coincides (up to the replacement of uridine nucleotides with thymine nucleotides) with the DNA sequence in the sense strand, i.e. in order to determine the desired genome region, it is sufficient to replace all **U** nucleotides with **T** nucleotides in the mRNA region constructed at the previous stage. Note that both DNA strands can act as a sense or an antisense strand for different transcripts. Thus, to construct the DNA subsequence which was further used at the analysis stage itself, we performed the following transformations of the microRNA:

1. invert nucleotides in the microRNA;
2. construct the nucleotide sequence complementary to the inverted microRNA;
3. replace all the occurrences of uridine with thymine in the obtained sequence.

**Fig. 2:**
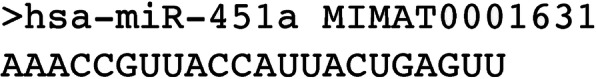
hsa-miR-451a sequence (miRBase entry for MI0001729)

**Fig. 3:**
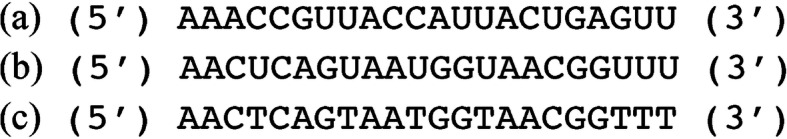
MicroRNA sequence transformations (hsa-miR-451a): **a** the original microRNA sequence, **b** the complementary mRNA, **c** the sequence with all uridine nucleotides substituted with thymine nucleotides, i.e. a DNA region corresponding to an mRNA complementary to the microRNA

Since the majority of the studies examined in the literature review demonstrate an increase in the level of miR-451a, we use it to illustrate the algorithm. The sequence of nucleotides comprising the 5′ chain of this microRNA is shown in Fig. 2. The output of the transformation algorithm and its intermediate stages are illustrated by Fig. 3.

### The analysis

The target gene prediction algorithm included 3 stages that were performed for each pair (microRNA; isolate):

1. Identify the intervals homologous to the inverted microRNA in the unannotated genome using the blastn tool (parameters: -task blastn-short -word size 7 -evalue N, where *N* ∈{100,1000}).
2. Locate the coordinates of the beginning and end of the homologous interval of the product of the target gene. At this stage we additionally used an annotated genome the sequences in which fully coincided with the unannotated sequenced used at the previous stage.
3. Generate a final report containing both information about the product of the target gene and other information obtained using the blastn tool during the first stage of the analysis: the coordinates of the homologous interval between the genome and the microRNA, DNA chain direction, mathematical expectation, reflecting the number in the genome of sequences similar to the obtained homology interval.

It is worth mentioning that a microRNA contains the so-called seed region, which, as a rule, consists of nucleotides lying in the [2, 8] interval; these sequence is completely or almost completely complementary to a subsequence of the target mRNA. Since the initial nucleotide sequence was inverted, the nucleotides complementary to the seed region occupies the position between [*len − end + 1;len − begin + 1*], e.g. nucleotides [14, 20] in the case of hsa-miR-451a. Thus, those sequences which have the best complementarity in this interval should have the greatest weight among all detected sequences.

## Results

We have analysed genomes of 15 *P. falciparum* isolates (3D7, 7G8, Dd2, GB4, CD01, GA01, GN01, HB3, IT, KE01, KH01, KH02, ML01, SN01, TG01) and 7 microRNAs (hsa-miR-451a, hsa-miR-223-3p, hsa-let-7b-5p, hsa-miR-223-5p, hsa-miR-150-5p, hsa-miR-486-5p, hsa-miR-106b-5p) (Table 1). The microRNAs had been selected according to the upregulation of their levels reported in literature. These observations are summarised in Table 2. It is worth mentioning that the level of the let-7a microRNA has been reported to be increased in two studies, namely, by Xue et al. [7] and Mantel et al. [16], but Sisquella et al. [15] has observed its decrease. One of the most important criteria for assessing the potential involvement of microRNAs in regulating the level of expression of *P. falciparum* genes and, as a consequence, their influence on the course of pathological processes in the patient with *P. falciparum* malaria, is the ability of these microRNAs to interact with cell adhesion proteins expressed by the parasite: PfEMP1, RIFIN, STEVOR.

**Table 1:** Homologous sequences of the *P. falciparum* 3D7 genome and hsa-miR-451a

	Sequence	Strand	***E***(***ξ***)	Gene
1	chr03, [8;22] – [914,782;914,796]	–	0.59	[‘WD repeat-containing protein, putative’]
2	chr14, [1;14] – [1,367,718;1,367,731]	+	2.3	[‘queuine tRNA-ribosyltransferase, putative’]
3	chr03, [7;19] – [959,691;959,703]	+	9.2	[‘conserved Plasmodium protein, unknown function’]
4	chr14, [6;18] – [384,208;384,220]	+	9.2	[‘CUGBP Elav-like family member 2, putative’]
5	chr14, [6;18] – [1,683,794;1,683,806]	+	9.2	[‘serine/threonine protein kinase, putative’]
6	chr13, [6;18] – [34,841;34,853]	–	9.2	[‘erythrocyte membrane protein 1, PfEMP1’]
7	chr13, [11;23] – [1,771,091;1,771,103]	–	9.2	[‘heat shock protein 110, putative’]
8	chr13, [6;18] – [2,342,648;2,342,660]	+	9.2	[‘conserved Plasmodium protein, unknown function’]
9	chr13, [2;14] – [2,824,580;2,824,592]	–	9.2	[‘Plasmodium exported protein, unknown function’]
10	chr11, [6;18] – [33,629;33,641]	–	9.2	[‘erythrocyte membrane protein 1, PfEMP1’]
11	chr11, [6;18] – [1,263,345;1,263,357]	–	9.2	[‘amino acid transporter, putative’]
12	chr11, [11;23] – [1,938,359;1,938,371]	–	9.2	[]
13	chr10, [6;18] – [192,361;192,373]	–	9.2	[‘WD repeat-containing protein, putative’]
14	chr09, [6;18] – [1,054,106;1,054,118]	–	9.2	[‘protein kinase, putative’]
15	chr08, [5;17] – [280,093;280,105]	–	9.2	[‘GTPase-activating protein, putative’]
16	chr07, [6;18] – [635,116;635,128]	–	9.2	[‘conserved Plasmodium protein, unknown function’]
17	chr07, [6;18] – [1,411,278;1,411,290]	+	9.2	[‘erythrocyte membrane protein 1 (PfEMP1), exon 2’]
18	chr06, [6;18] – [11,933;11,945]	+	9.2	[‘erythrocyte membrane protein 1, PfEMP1’]
19	chr04, [6;18] – [46,533;46,545]	–	9.2	[‘erythrocyte membrane protein 1, PfEMP1’]
20	chr04, [6;18] – [947,120;947,132]	–	9.2	[‘erythrocyte membrane protein 1, PfEMP1’]
21	chr04, [6;18] – [959,018;959,030]	–	9.2	[‘erythrocyte membrane protein 1, PfEMP1’]
22	chr02, [6;18] – [47,635;47,647]	+	9.2	[]
23	chr02, [6;18] – [910,131;910,143]	+	9.2	[‘erythrocyte membrane protein 1 (PfEMP1), exon 2’]
24	chr01, [6;18] – [43,297;43,309]	–	9.2	[‘erythrocyte membrane protein 1, PfEMP1’]

**Table 2:** Comparison of microRNA expression levels in *P. falciparum* and *P. berghei* malaria (amended from Chamnanchanunt et al. [14]

***Plasmodium*** species	MicroRNA level increase	MicroRNA level decrease
*P. falciparum* culture [24]	significant increase of miR-451 in erythrocytes	–
*P. falciparum* in erythrocytes [7]	invaded erythrocytes contain miR-451, let-7b, miR-16, miR-91, miR-142, miR-144, let-7a, let-7f, miR-92, miR-106	–
*P. falciparum* in vitro culture, HbAS and HbCC erythrocytes [11]	miR-451, miR-223	–
*P. falciparum* 3D7 culture [16]	erythrocytes contain miR-451a, let-7b, let-7a, miR-106b, miR-15b	–
*P. falciparum* culture, isolates CS2eBsdGFP, 3D7edhfrGFP, NF54, CS2 3D7 [15]	–	invaded erythrocytes is decreased, most significantly: miR-19b, miR-4732, let-7a, miR-16, miR-183, miR-18a, miR148b
*P. falciparum* in erythocytes [19]	the invaded erythrocytes contain 305 kinds of human microRNAs, predominantly miR-451a, miR-486-5p, miR-92a-3p, miR-103a-3p	–
*P. berghei* ANKA [25]	let-7i, miR-27a, miR-150	–
*P. berghei* ANKA [26]	miR-146a, miR-486, miR-215, miR-21, miR-150	miR-193b, miR-467a
*P. berghei* ANKA [27]	miR-19a-3p, miR-19b-3p, miR-142-3p, miR-223-3p, miR-540-5p	–

**Fig. 4:**
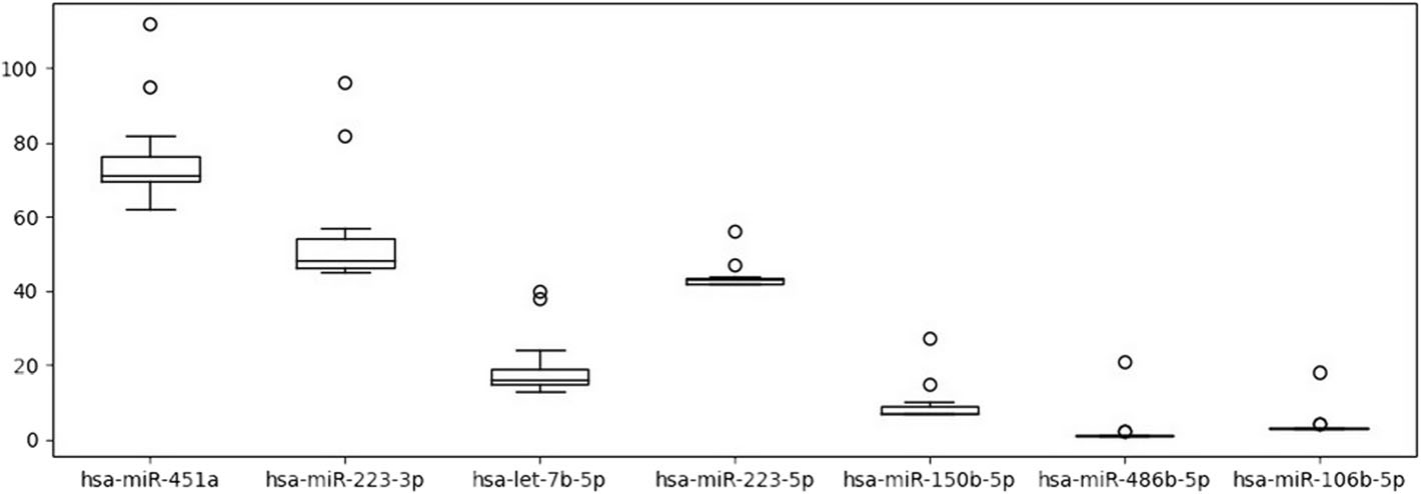
The number of identified potential interactions between the *P. falciparum* mRNAs and the human microRNAs (total)

One of the most important criteria in the assessment of the potential importance of a microRNA in *P. falciparum* gene expression regulation and towards the prognosis of the disease outcome was its ability to interact with mRNAs encoding cell adhesion proteins expressed by the parasite, namely, PfEMP1, RIFIN and STEVOR. Table 3 and Fig. 4 summarise average numbers of the identified homologous intervals between the analysed microRNAs and the genomes of all *P. falciparum* isolates, hence demonstrating the potential ability of these microRNAs to interact with the mRNAs by the corresponding genome intervals. We analysed genes encoding cell adhesion proteins (PfEMP1, RIFIN, STEVOR; excluding pseudogenes) separately due to their importance in the pathogenesis of malaria, as well as all genes encoding proteins and RNA in general.

**Table 3:** The number of identified potential interactions between the *P. falciparum* mRNAs and the human microRNAs

	MicroRNA	Number of interactions		
		With cell adhesion proteins	With genes	Total
1	hsa-miR-451a	19.2 (0.25)	75.8 (0.83)	91.07
2	hsa-miR-223-3p	16.87 (0.31)	54.47 (0.39)	139.47
3	hsa-let-7b-5p	6.6 (0.34)	19.53 (0.44)	44.6
4	hsa-miR-223-5p	1.6 (0.04)	43.87 (0.60)	73.4
5	hsa-miR-150-5p	1.6 (0.17)	9.4 (0.56)	16.93
6	hsa-miR-486-5p	0.87 (0.35)	2.47 (0.54)	4.6
7	hsa-miR-106b-5p	0.73 (0.18)	4.13 (0.32)	12.87

The potential ability of different *P. falciparum* isolates to interact with the human microRNAs was analysed in more detail; the results of this analysis are summarised in Tables 4, 5, 6, 7, 8, 9 and 10. The analysis of the target prediction results has revealed the following patterns:

1. The number of homologous intervals with the same microRNA significantly differs between various *P. falciparum* isolates.
2. In most cases, the majority of homologous intervals are located within genes encoding PfEMP1. The number of homologous intervals with RIFIN genes is significantly lower and close to zero in case of STEVOR genes. In none of the cases under consideration the number of homologous intervals with PfEMP1 pseudogenes exceeded those with genes encoding the other cell adhesion proteins.

**Table 4:** The numbers of homologous sequences within the genomes of various *P. falciparum* isolates indicating potential interactions between the encoded mRNAs and the hsa-miR-451a microRNA

	Isolate	PfEMP1	RIFIN	STEVOR	PfEMP1 (pseudogene)	Total
1	TG01	28	11	7	2	112
2	ML01	22	9	8	4	95
3	GN01	22	1	0	4	82
4	Dd2	21	0	0	0	75
5	GB4	18	1	0	0	75
6	KH01	16	2	0	1	71
7	3D7	16	0	0	0	71
8	SN01	15	4	0	2	78
9	KH02	15	0	0	0	68
10	IT	14	1	0	0	71
11	GA01	14	0	0	0	69
12	HB3	13	0	0	0	70
13	KE01	12	0	1	2	72
14	CD01	11	1	0	4	66
15	7G8	3	2	0	1	62

**Table 5:** The numbers of homologous sequences within the genomes of various *P. falciparum* isolates indicating potential interactions between the encoded mRNAs and the hsa-miR-223-3p microRNA

	Isolate	PfEMP1	RIFIN	STEVOR	PfEMP1 (pseudogene)	Total
1	ML01	23	16	11	6	96
2	TG01	21	8	3	0	82
3	GN01	19	1	0	0	57
4	CD01	19	0	0	0	54
5	KH01	18	0	0	0	53
6	SN01	15	1	0	1	54
7	3D7	15	0	0	0	51
8	IT	13	0	0	0	48
9	KH02	11	0	0	0	47
10	GA01	11	0	0	0	46
11	Dd2	10	1	0	0	46
12	7G8	10	0	0	0	46
13	HB3	9	0	0	0	46
14	KE01	9	0	0	0	45
15	GB4	8	1	0	0	46

**Table 6:** The numbers of homologous sequences within the genomes of various *P. falciparum* isolates indicating potential interactions between the encoded mRNAs and the hsa-let-7b-5p microRNA

	Isolate	PfEMP1	RIFIN	STEVOR	PfEMP1 (pseudogene)	Total
1	TG01	13	8	6	1	40
2	ML01	5	7	5	4	38
3	SN01	3	7	0	1	24
4	KE01	3	4	0	0	19
5	KH01	3	4	0	0	19
6	CD01	2	4	0	0	18
7	KH02	1	3	0	0	16
8	Dd2	1	2	0	1	16
9	GB4	1	2	0	0	15
10	GA01	0	4	0	0	16
11	3D7	0	3	0	0	16
12	GN01	0	3	0	0	15
13	IT	0	2	0	0	14
14	HB3	0	2	0	0	14
15	7G8	0	1	0	0	13

**Table 7:** The numbers of homologous sequences within the genomes of various *P. falciparum* isolates indicating potential interactions between the encoded mRNAs and the hsa-miR-223-5p microRNA

	Isolate	PfEMP1	RIFIN	STEVOR	PfEMP1 (pseudogene)	Total
1	TG01	4	3	3	1	56
2	IT	2	0	0	0	44
3	ML01	1	2	1	1	47
4	KH01	1	1	0	0	43
5	CD01	1	0	1	0	44
6	KE01	1	0	0	0	43
7	SN01	1	0	0	0	43
8	GN01	1	0	0	0	43
9	HB3	1	0	0	0	42
10	3D7	0	0	0	0	43
11	Dd2	0	0	0	0	42
12	GA01	0	0	0	0	42
13	GB4	0	0	0	0	42
14	KH02	0	0	0	0	42
15	7G8	0	0	0	0	42

**Table 8:** The numbers of homologous sequences within the genomes of various *P. falciparum* isolates indicating potential interactions between the encoded mRNAs and the hsa-miR-150b-5p microRNA

	Isolate	PfEMP1	RIFIN	STEVOR	PfEMP1 (pseudogene)	Total
1	ML01	5	5	3	3	27
2	TG01	3	3	3	1	15
3	IT	0	1	0	0	9
4	HB3	0	1	0	0	9
5	SN01	0	0	0	1	10
6	7G8	0	0	0	0	8
7	KE01	0	0	0	0	7
8	3D7	0	0	0	0	7
9	Dd2	0	0	0	0	7
10	CD01	0	0	0	0	7
11	GA01	0	0	0	0	7
12	GB4	0	0	0	0	7
13	KH01	0	0	0	0	7
14	GN01	0	0	0	0	7
15	KH02	0	0	0	0	7

**Table 9:** The numbers of homologous sequences within the genomes of various *P. falciparum* isolates indicating potential interactions between the encoded mRNAs and the hsa-miR-486b-5p microRNA

	Isolate	PfEMP1	RIFIN	STEVOR	PfEMP1 (pseudogene)	Total
1	ML01	5	3	3	3	21
2	KH02	1	0	0	0	2
3	KH01	0	1	0	0	2
4	KE01	0	0	0	0	1
5	3D7	0	0	0	0	1
6	TG01	0	0	0	0	1
7	Dd2	0	0	0	0	1
8	SN01	0	0	0	0	1
9	IT	0	0	0	0	1
10	CD01	0	0	0	0	1
11	GA01	0	0	0	0	1
12	GB4	0	0	0	0	1
13	GN01	0	0	0	0	1
14	7G8	0	0	0	0	1
15	HB3	0	0	0	0	1

**Table 10:** The numbers of homologous sequences within the genomes of various *P. falciparum* isolates indicating potential interactions between the encoded mRNAs and the hsa-miR-106b-5p microRNA

	Isolate	PfEMP1	RIFIN	STEVOR	PfEMP1 (pseudogene)	Total
1	TG01	2	3	5	0	18
2	KH01	1	0	0	0	4
3	ML01	0	0	0	0	4
4	KE01	0	0	0	0	3
5	3D7	0	0	0	0	3
6	Dd2	0	0	0	0	3
7	SN01	0	0	0	0	3
8	IT	0	0	0	0	3
9	CD01	0	0	0	0	3
10	GA01	0	0	0	0	3
11	GB4	0	0	0	0	3
12	GN01	0	0	0	0	3
13	KH02	0	0	0	0	3
14	7G8	0	0	0	0	3
15	HB3	0	0	0	0	3

The numbers of homologous intervals with hsa-miR-451a are maximal for all isolates; these values are reflected in the last column. Among the analysed genes encoding cell adhesion proteins, as indicated by columns 3–6, the majority of potential interactions are expected to happen with mRNAs encoding the PfEMP1 protein, and hence this protein is expected to be particularly affected by the RNA interference-like mechanism. With minor exceptions, the numbers representing potential interactions with specific cell adhesion proteins for hsa-miR-451a are higher in comparison with all the other microRNAs under consideration. Interestingly, the total number of homologous intervals with this microRNA differs almost twice between the isolates; the separate quantities calculated for specific genes encoding cell adhesion proteins differ even more significantly: the proportion of homologous intervals with PfEMP1 genes within the *P. falciparum* TG01 isolate genome is higher by an order of magnitude than the one in the *P. falciparum* 7G8 isolate.

## Discussion

The bioinformatics research indicates that the expression of *P. falciparum* genes might be regulated by human microRNAs using a non-canonical mechanism similar to RNA interference. We have found that the genome of *P. falciparum* contains genes encoding mRNA potentially capable of forming duplexes with human microRNAs. Taking into consideration the previously demonstrated fact of transport of the RISC elements into the organism of the parasite, this result may indicate the possibility of RNA interference in *P. falciparum* and regulation of gene expression through human microRNAs.

The topic to which this work is devoted is not quite well covered in the literature. To our knowledge, only two similar works exist: one is by LaMonte et al. [11] and the other one is by Dandewad et al. [12]. Interestingly, LaMonte et al. [11] in their work have suggested that the mechanism of the *P. falciparum* gene suppression is completely different and is based on the formation of covalent bonds between imported human microRNAs and 5′ ends of *P. falciparum* mRNAs similar to trans-splicing. It is worth mentioning that we have found no other works that would support this hypothesis. Thus, the pathway according to which human microRNA enter the parasite and form hydrogen bonds with complementary sequences in plasmodial mRNAs or non-coding RNAs seems more feasible. However, the authors also consider miR-451a as the major factor of *P. falciparum* gene expression deregulation. Besides that, the novelty of this study is provided by the fact that we have found no works in which the researchers would specifically emphasise the role of the cell adhesion proteins of *P. falciparum* as a potential target for human microRNAs.

## Conclusions

As a result of the analysis, it has been found that a significant proportion (up to 1/3) of all mRNAs with which the investigated human microRNAs can form complementary bonds are mRNAs encoding cell adhesion proteins of *P. falciparum*. This fact allows us to consider this phenomenon to be an antiparasitic protective mechanism of the human. The significant numerical superiority of the number of binding sites for the hsa-miR-451a and hsa-miR-223-3p microRNAs that was revealed as a result of the analysis correlates with the increase in the level of these microRNAs in patients with malaria described in the literature. Similarly, the observed patterns in the number of binding sites in mRNAs encoding adhesion proteins correspond to the fact that sequestration of red blood cells and rosette formation can be mediated by the PfEMP1, RIFIN, and STEVOR proteins, with PfEMP1 playing the leading role in these processes. In addition, the analysis of the genomes of various *P. falciparum* isolates has shown significant differences in the number of possible target mRNAs for human microRNAs, which may serve as a diagnostic criterion for determining the strain of *P. falciparum* and for prediction of the further course of malaria.

The main direction of further work is experimental verification of the aforementioned hypotheses using molecular methods, namely, microRNA sequencing to determine microRNA profiles in different cases and quantitative PCR to explore relationships between the human microRNA and plasmodial RNA levels. Besides, it is essential to research the existence of similar mechanisms in other species belonging to the *Plasmodium* genus and to determine the possible effect of microRNA of the macroorganism on the course of other forms of malaria.

## Data Availability

All data will be available on request.

## Abbreviations

ATF2: Activating transcription factor 2
CAV-1: Caveolin-1
DNA: Deoxyribonucleic acid
IL-1: Interleukin-1
IL-6: Interleukin-6
mRNA: Messenger RNA
ncRNA: Non-coding RNA
PfEMP1: *Plasmodium falciparum* erythrocyte membrane protein 1
piRNA: Piwi-interacting RNA
PTGS: Posttranscripional gene silencing
RIFIN: Repetitive interspersed family
RISC: Reduced-instruction-set computing
RNA: Ribonucleic acid
rRNA: Ribosomal RNA
siRNA: Small interfering RNAs
snoRNA: Small nucleolar RNA
snRNA: Small nuclear RNA
STEVOR: Subtelomeric variable open reading frame
tRNA: Transfer RNA
vaultRNA: Vault RNA
VCAM-1: Vascular cell adhesion molecule 1
Y-RNA: Small non-coding RNAs
